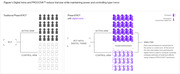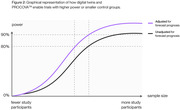# Assessment of AI‐generated digital twin (DT) methodology on reduction of treatment effect variance and potential clinical trial sample size saving using a Phase 2 trial dataset from patients with Alzheimer’s disease (AD)

**DOI:** 10.1002/alz.090685

**Published:** 2025-01-09

**Authors:** Deli Wang, Hana Florian, Shau Yu Lynch, Weining Z Robieson, Li Wang, Sheng Zhong, Yunzhao Xing, Run Zhuang, Coco Kusiak, Stefanie Millar, Michelle Turner, Jess Ross, Jonathan R. Walsh, Katelyn Begany, Ole Graff

**Affiliations:** ^1^ AbbVie Inc., North Chicago, IL USA; ^2^ Unlearn.AI, San Francisco, CA USA

## Abstract

**Background:**

In Alzheimer’s Disease (AD) trials, clinical scales are used to assess treatment effect in patients. Minimizing statistical uncertainty of trial outcomes is an important consideration to increase statistical power. Machine learning models can leverage baseline data to create AI‐generated digital twins – individualized predictions (or prognostic scores) of how each patient’s clinical outcomes may change during a trial assuming they received placebo. Incorporating prognostic scores into trial design and analysis as a covariate increases statistical power, or reduces sample size, in Phase 2 and 3 trials (Figures 1/2). We assessed these properties using data from a Phase 2 clinical trial of tilavonemab in patients diagnosed with early AD (NCT02880956) and digital twin (DT) methodology (PROCOVA^TM^).

**Method:**

In a double‐blind, Phase 2 trial (AWARE), 453 patients aged 55‐85 years with early AD (met NIA‐AA clinical criteria for mild cognitive impairment or probable AD), were randomized to receive placebo or 1 of 3 doses of tilavonemab (1:1:1:1 ratio) over a 96‐week treatment period. Prognostic scores were produced for the change from baseline (Δ) in Clinical Dementia Rating Scale Sum of Boxes (CDR‐SB) and the Δ in AD Assessment Scale‐Cognitive Subscale 14 (ADAS‐Cog 14). Sample size savings were calculated from partial Pearson correlations (controlled for treatment) between prognostic scores and trial outcomes. Variability reductions were assessed using a covariance modelling approach that adjusts for the prognostic score (PROCOVA^TM^).

**Result:**

For Δ CDR‐SB and Δ ADAS‐Cog 14 at Week 96, standard deviations of the prognostic scores were lower than the trial’s outcomes. Partial correlation coefficients were moderate for both Δ CDR‐SB (p = 0.360) and Δ ADAS‐Cog 14 (p = 0.305) at Week 96. Total residual variance for both outcomes was reduced by ∼11% with DT methodology compared to an unadjusted model.

**Conclusion:**

Depending on correlations between prognostic scores and actual trial outcomes, a potential overall sample size reduction of 5‐10% could be achieved using DT methodology (PROCOVA^TM^) while maintaining statistical power, based on Δ CDR‐SB and Δ ADAS‐Cog 14 in the AWARE study. Sample size savings could enable shortening of the recruitment period and reduce the number of patients on placebo, encouraging greater patient participation.